# Influence of viral hepatitis status on prognosis in patients undergoing hepatic resection for hepatocellular carcinoma: a meta-analysis of observational studies

**DOI:** 10.1186/1477-7819-9-108

**Published:** 2011-09-21

**Authors:** Yanming Zhou, Xiaoying Si, Lupeng Wu, Xu Su, Bin Li, Zhiming Zhang

**Affiliations:** 1 Department of Hepato-Biliary-Pancreato-Vascular Surgery, the First affiliated Hospital of Xiamen University, Xiamen, China; 2 Department of Blood Transfution, the First affiliated Hospital of Xiamen University, Xiamen, China; 3 Cancer Center, the First affiliated Hospital of Xiamen University, Xiamen, China

**Keywords:** Hepatocellular carcinoma, Viral infection, Hepatitis B, Hepatitis C, Prognosis

## Abstract

**Background:**

The influence of viral hepatitis status on prognosis in patients undergoing hepatic resection for hepatocellular carcinoma (HCC) remains a matter of debate. This study is a meta-analysis of the available evidence.

**Methods:**

A literature search was performed to identify comparative studies reporting postoperative survival of HCC in different types of viral hepatitis. Pooled odds ratios (OR) and weighted mean differences (WMD with 95% confidence intervals (95% CI) were calculated using either the fixed effects model or random effects model.

**Results:**

Twenty studies matched the selection criteria and reported on 4744 subjects, of whom 2008 in the HBV-positive (B-HCC) group, 2222 in the HCV-positive (C-HCC) group, and 514 in the hepatitis B- and C-negative (NBNC-HCC). Meta-analysis showed that patients with HBV or HCV infection had a worse 5-year disease-free survival when compared to patients with NBNC-HCC (respectively: OR: 0.39, 95% CI: 0.28 to 0.53, P < 0.001; WMD: 0.37, 95% CI: 0.22 to 0.64, P < 0.001). There was a tendency toward higher 5-year overall survival rates in the NBNC-HCC group compared to those in the other two groups, although these differences were not statistically significant. Both the 5-year overall survival and disease-free survival were not different among the B-HCC and C-HCC groups.

**Conclusions:**

Patients with positive serology for hepatitis B or C undergoing resection for HCC had a poor prognosis compared to patients with negative serology.

## Background

Hepatocellular carcinoma (HCC) is the fifth most common cancer in the world, responsible for 500,000 deaths globally every year [1]. Chronic viral hepatitis and liver cirrhosis related to hepatitis B virus (HBV) and hepatitis C virus (HCV) infections represent the major known risk factors for HCC. A review of the literature reveals that 75% to 80% of cases of HCC are attributable to persistent viral infections with either HBV (50%-55%) or HCV (25%-30%) [2]. Nevertheless, some patients with HCC are dually infected, whereas others are negative for both HBV and HCV [3-7].

Hepatic resection is widely accepted as the treatment of choice for HCC. With regard to surgery, it is important to determine whether or not the prognosis after resection differ according to the viral status. So far, the influence of viral status on prognosis for patients with HCC treated by resection remains controversial. For example, Yamanaka *et al*. [3] reported that the disease-free and overall survival rates of hepatitis B- and C-negative group were better than those of viral infections groups. In contrast, Pawlik *et al*. [5] reported that the presence of viral hepatitis did not significantly affect the survival rate.

Meta-analysis can be used to evaluate the existing literature in both a qualitative and quantitative way by comparing and integrating the results of different studies and taking into account variations in characteristics that can influence the overall estimate of the outcome of interest [8]. This study uses metaanalytical techniques to evaluate the influence of viral hepatitis status on prognosis in patients with HCC treated by surgery.

## Methods

### Study Selection

Infection with HBV was defined as positivity for hepatitis B surface antigen (HBsAg) or for anti-hepatitis B core antibody. Infection with HCV was defined as positivity for serum anti-HCV antibody (HBcAb). Therefore, patients were divided into four groups: HBV-positive (B-HCC), HCV-positive (C-HCC), dual hepatitis B- and C positive (BC-HCC), and hepatitis B- and C-negative (NBNC-HCC). A MEDLINE, EMBASE, OVID, and Cochrane database search was performed on all studies reporting postoperative survival between four groups. The following Mesh search headings were used: "hepatitis B virus," "hepatitis C virus," "hepatocellular carcinoma," "survival rate," "liver resection," and "hepatectomy". Only studies on humans and in English language were considered for inclusion. Reference lists of all retrieved articles were manual searched for additional studies.

### Data Extraction

Two reviewers (LW and XS, respectively) independently extracted the following parameters from each study: first author, year of publication, study population characteristics, study design, inclusion and exclusion criteria, number of patients with different preoperative viral status, male: female ratio. All relevant text, tables and figures were reviewed for data extraction. Discrepancies between the two reviewers were resolved by discussion and consensus.

### Criteria for Inclusion and Exclusion

For inclusion in the meta-analysis, a study had to fulfill the following criteria: 1) evaluate the influence of viral hepatitis status on prognosis in HCC patients undergoing hepatic resection; 2) report on at least one of the outcome measures mentioned below; 3) In dual (or multiple) studies were reported by the same institution and/or authors, either the one of higher quality or the most recent publication was included in the analysis.

Abstracts, letters, editorials and expert opinions, reviews without original data, case reports and studies lacking control groups were excluded. The following studies were also excluded: 1) those with no clearly reported outcomes of interest; 2) those evaluating patients with other types of malignant liver tumors and did not contain a distinct group of patients with HCC; or (3) those including patients undergoing palliative treatment (noncurative surgical intent).

### Outcomes of Interest

Primary outcomes of interest were 5-year overall and disease-free survival after resection. Secondary outcomes of interest were clinicopathologic features.

### Statistical Methods

The meta-analysis was performed using the Review Manager (RevMan) software, version 4.2.7 (The Cochrane Collaboration, Software Update, Oxford). We analysed dichotomous variables using estimation of odds ratios (OR) with a 95% confidence interval (95% CI), and continuous variables using weighted mean difference (WMD) with a 95% CI. The overall effect was tested using Z scores, with significance being set at *P *< 0.05. Pooled effect was calculated using either the fixed effects model or random effects model. Heterogeneity was evaluated by χ^2 ^and I^2^. In the absence of statistically significant heterogeneity, the fixed-effect method was used to combine the results. When heterogeneity was confirmed (*P *≤ 0.10), the random-effect method was used [9].

## Results

### Selection of Studies

The search strategy initially generated 38 studies [3-7,10-42]. Of these studies, 18 were excluded for various reasons: 11 including patients with unresectable lesions [6,7,10-18], four without survival information [19-21]. Three were published by the same team with overlapping study populations [23-25]. Finally, a total of 20 studies published between 1995 and 2011 matched the inclusion criteria and were therefore included [3-5,27-42].

The patients with BC-HCC were too small in number and so were not separately analyzed in many studies. Only seven of 20 studies reported 186 cases of such patients in current review [3-5,27-29]. To avoid high bias-risk of publication, we did not perform an analysis of BC-HCC group. Therefore, 4744 patients were included in the meta-analysis, of whom 2008 in the B-HCC group, 2222 in the C-HCC group, and 514 in the NBNC-HCC group. The median or mean (range) duration for the entire cohort of patients in 11 studies providing data on follow-up ranged from 20.3 to 132 months. In two manuscripts, Ahmad *et al*. [35] and Sasaki *et al*. [40] reported the data of subsets of patients. The characteristics of these 20 studies are summarized in Table 1.

**Table 1:** Baseline characteristics of studies included in the meta-analysis

Author	Year	Country	Group	No. of patients	Male/ Female	Mean age (years)	Mean follow-up (months)
Takenaka [30]	1995	Japan	B-HCC C-HCC	30 96	22/8 77/19	57.0 ± 9.4 61.7 ± 6.9	-- --
Miyagawa [31]	1996	Japan	B-HCC C-HCC NBNC	32 124 19	21/11 96/28 15/4	52.1 ± 12.4 63.9 ± 7.0 62.2 ± 11.8	-- -- --
Yamanaka [3]	1997	Japan	B-HCC C-HCC NBNC	27 151 20	24/3 125/26 18/2	51 ± 10 63 ± 6.3 63 ± 6.4	-- -- --
Wu [26]	1999	Taiwan	B-HCC C-HCC NBNC	131 70 40	110/21 56/14 29/11	54.3 ± 1.1 64.1 ± 1.1 68.9 ± 1.9	34.5*&
Shiraishi [32]	1999	Japan	B-HCC C-HCC NBNC	11 21 12	-- -- --	54.0 ± 3.2 62.0 ± 1.8 63.0 ± 4.1	-- -- --
Lee [4]	2000	Taiwan	B-HCC C-HCC NBNC	133 66 30	112/21 48/18 20/10	49.4 ± 12.7 61.7 ± 9.2 54.3 ± 13.3	23.5 ± 16.3 &
Noguchi [33]	2000	Japan	B-HCC C-HCC NBNC	44 232 13	34/10 172/60 12/1	51.6 ± 8.4 65.0 ± 7.0 60.9 ± 6.7	-- -- --
Roayaie [34]	2000	United States	B-HCC C-HCC	21 24	10/11 17/7	54.3 ± 15.3 63.4 ± 8.5	20.3*&
Ahmad [35]	2001	United States	B-HCC C-HCC NBNC	18 44 15	13/5 34/10 6/9	60 61 63	30* 27* 33*
Chen [36]	2001	Taiwan	B-HCC C-HCC	211 59	190/21 47/12	57.6 ± 12.7 66.9 ± 8.2	-- --
Wakai [37]	2003	Japan	B-HCC C-HCC NBNC	32 55 24	20/12 46/9 18	52.5 (16-77)* 64 (46-78) 68 (45-79)	75*&
Pawlik [5]	2004	Multi center	B-HCC C-HCC NBNC	163 79 126	137/26 48/31 90/36	60* 60 51	33*&
Uchiyama [39]	2005	Japan	B-HCC C-HCC NBNC	25 72 24	18/7 48/24 18/6	54 ± 10 64 ± 9 65 ± 8	-- -- --
Yokoi [38]	2005	Japan	B-HCC C-HCC NBNC	25 116 13	19/6 95/21 10/3	57 (32-74)* 64 (46-85) 58 (28-72)	-- -- --
Sasaki [40]	2006	Japan.	B-HCC C-HCC	66 351	49/17 268/83	> 65 (n = 5) > 65 (n = 114)	132* 121.2*
Li [27]	2007	China	B-HCC C-HCC NBNC	251 75 54	212/39 62/13 44/10	51.2 ± 4.2 63.2 ± 7.3 67.1 ± 5.7	48.3* &
Nanashima [28]	2007	Japan	B-HCC C-HCC NBNC	76 124 29	61/15 99/25 21/8	59 ± 11 67 ± 7 65 ± 8	-- -- --
Kondo [41]	2008	Japan	B-HCC C-HCC NBNC	78 127 60	58/20 94/33 43/17	54.7 ± 11.6 67.2 ± 6.7 67.9 ± 10.3	26*&
Cescon [29]	2009	Italy	B-HCC C-HCC NBNC	25 130 35	24/1 90/40 30/5	60.2 ± 9.8 65.2 ± 8.1 64.2 ± 9.1	30*&
Kao [42]	2011	Taiwan	B-HCC C-HCC	609 206	516/93 147/59	56.3 ± 13.5 67.2 ± 9.1	40.6*&

HCC = hepatocellular carcinoma; B-HCC = hepatitis B-related hepatocellular carcinoma; C-HCC = hepatitis C-related hepatocellular carcinoma; NBNC-HCC = no infection of HBV or HCV related hepatocellular carcinoma; * = median; & = entire group

### Patients Characteristics

Results from overall meta-analysis are outlined in Table 2.

**Table 2:** Results of a meta-analysis

Outcome of interest	No. of studies	No.of patients	Results	OR/WMD	95% CI	*P*-value	I^2 ^(%)
Patients characteristics							
Age (years)							
B-HCC versus C-HCC	15 [3,4,26-34,36,39,41,42]	3281	B-HCC = 54.4 ± 9.2, C-HCC = 64.3 ± 6.8	-10.11	-11.14, -9.09	< 0.001	65.3
B-HCC versus NBNC-HCC	11 [3,4,26,27,29,31-33,39,41]	1169	B-HCC = 53.7 ± 8.5, NBNC-HCC = 63.7 ± 7.7	-10.42	-12.72, -8.12	< 0.001	86.1
C-HCC versus NBNC-HCC	11 [3,4,26,27,29,31-33,39,41]	1528	C-HCC = 64.2 ± 6.4, NBNC-HCC = 63.7 ± 7.7	0.08	-2.18, 2.38	0.95	88.2
Male							
B-HCC versus C-HCC	19 [3-5,26-31,33-42]	4198	B-HCC = 82.6%, C-HCC = 75.8%	1.19	0.89, 1.60	0.24	61.2
B-HCC versus NBNC-HCC	14 [3-5,26-29,31,33,35,37-39,41]	1562	B-HCC = 81.4%, NBNC-HCC = 74.5%	1.43	1.10, 1.86	0.008	16.3
C-HCC versus NBNC-HCC	14 [3-5,26-29,31,33,35,37-39,41]	1967	C-HCC = 75.9%, NBNC-HCC = 74.5%	0.96	0.74, 1.23	0.74	31
Liver function							
Serum ALT level (IU/l)							
B-HCC versus C-HCC	11 [3,4,27,29,30,32-34,36,39,41]	1909	B-HCC = 56.4 ± 44.8, C-HCC = 76.9 ± 47.6	-16.84	-21.02, -12.65	< 0.001	23.4
B-HCC versus NBNC-HCC	8 [3,4,27,29,32,33,39,41]	842	B-HCC = 56.7 ± 55.9, NBNC-HCC = 39.6 ± 31.1	15.30	4.59, 26.01	0.005	73.9
C-HCC versus NBNC-HCC	8 [3,4,27,29,32,33,39,41]	1122	C-HCC = 74.1 ± 43.8, NBNC-HCC = 39.6 ± 31.1	34.41	23.75, 45.08	< 0.001	84.9
Serum AST level (IU/l)							
B-HCC versus C-HCC	8 [3,4,29,30,32,33,39,41]	842	B-HCC = 60.0 ± 56.7, C-HCC = 70.8 ± 38.1	-13.17	-22.29, -4.05	0.005	61.5
B-HCC versus NBNC-HCC	7 [3,4,29,32,33,39,41]	537	B-HCC = 61.0 ± 55.9, NBNC-HCC = 43.8 ± 25.5	13.06	0.13, 26.00	0.05	72.8
C-HCC versus NBNC-HCC	7 [3,4,29,32,33,39,41]	993	C-HCC = 69.9 ± 37.7, NBNC-HCC = 43.8 ± 25.5	24.87	18.94, 30.79	< 0.001	56.5
Serum albumin level (g/dl)							
B-HCC versus C-HCC	10 [3,27,29-31,33,34,36,39,41]	1834	B-HCC = 3.93 ± 0.48, C-HCC = 3.69 ± 0.48	0.23	0.08, 0.38	0.002	87.4
B-HCC versus NBNC-HCC	7 [3,27,29,31,33,39,41]	707	B-HCC = 3.91 ± 0.45, NBNC-HCC = 3.94 ± 0.48	-0.07	-0.15, 0.00	0.07	36.4
C-HCC versus NBNC-HCC	7 [3,27,29,31,33,39,41]	1136	C-HCC = 3.61 ± 0.47, NBNC-HCC = 3.94 ± 0.48	-0.29	-0.53, -0.05	0.002	89.7
ICG R15 (%)							
B-HCC versus C-HCC	10 [3,4,26,31-33,36,39,41]	1740	B-HCC = 12.9 ± 7.8, C-HCC = 20.4 ± 9.1	-6.58	-8.3, -4.87	< 0.001	78.9
B-HCC versus NBNC-HCC	8 [3,4,26,31-33,39,41]	699	B-HCC = 12.8 ± 7.5, NBNC-HCC = 13.9 ± 7.7	-0.74	-1.77, -0.30	0.16	21.3
C-HCC versus NBNC-HCC	8 [3,4,26,31-33,39,41]	1081	C-HCC = 21.0 ± 9.0, NBNC-HCC = 13.9 ± 7.7	5.92	3.85, 7.99	< 0.001	74.3
Child's grade A							
B-HCC versus C-HCC	9 [4,5,27,28,32,35,38,40,42]	2434	B-HCC = 88.3%, C-HCC = 80.8%	1.68	1.25, 2.25	< 0.001	34.9
B-HCC versus NBNC-HCC	7 [4,5,27,28,32,35,38]	956	B-HCC = 79.4%, NBNC-HCC = 80.6%	1.31	0.87, 1.98	0.20	0
C-HCC versus NBNC-HCC	7 [4,5,27,28,32,35,38]	804	C-HCC = 78.4%, NBNC-HCC = 80.6%	0.69	0.46, 1.05	0.08	1.1
Serum T-Bil level (mg/dL)							
B-HCC versus C-HCC	9 [4,27,29-31,33,36,39,41]	1579	B-HCC = 0.91 ± 0.47, C-HCC = 1.23 ± 0.83	-0.14	-0.27, -0.01	0.03	80.1
B-HCC versus NBNC-HCC	6 [4,27,29,31,39,41]	766	B-HCC = 0.92 ± 0.47, NBNC-HCC = 0.87 ± 0.49	0.06	-0.16, 0.28	0.60	90.3
C-HCC versus NBNC-HCC	6 [4,27,29,31,39,41]	816	C-HCC = 1.18 ± 0.76, NBNC-HCC = 0.87 ± 0.49	0.25	-0.02, 0.52	0.07	90.6
Serum platelet count (×10^3^/mm)							
B-HCC versus C-HCC	7 [27,29,30,33,34,39,41]	1230	B-HCC = 166.6 ± 85.0, C-HCC = 137.5 ± 66.9	24.47	1.24, 47.7	0.04	82.1
B-HCC versus NBNC-HCC	5 [27,29,33,39,41]	609	B-HCC = 156.8 ± 73.4, NBNC-HCC = 192.2 ± 72.4	-28.88	-41.93, -15.83	< 0.001	30.9
C-HCC versus NBNC-HCC	5 [27,29,33,39,41]	822	C-HCC = 138.2 ± 66.6, NBNC-HCC = 192.2 ± 72.4	-50.43	-75.13, -25.72	< 0.001	74.8
Tumor characteristics							
Size (cm)							
B-HCC versus C-HCC	10 [3,26,27,29,30,33,34,36,39,41]	1879	B-HCC = 5.4 ± 2.5, C-HCC = 4.0 ± 2.1	1.32	0.38, 2.27	0.006	98.4
B-HCC versus NBNC-HCC	7 [3,26,27,29,33,39,41]	827	B-HCC = 5.1 ± 2.5, NBNC-HCC = 5.3 ± 2.6	-0.02	-0.94, 0.00	0.97	96.5
C-HCC versus NBNC-HCC	7 [3,26,27,29,33,39,41]	1103	C-HCC = 3.8 ± 2.2, NBNC-HCC = 5.3 ± 2.6	-0.86	-1.27, -0.45	< 0.001	78.1
Coexisting cirrhosis							
B-HCC versus C-HCC	15 [3,4,26-32,34-38,40-42]	3623	B-HCC = 53.4%, C-HCC = 65.7%	0.71	0.54, 0.92	0.01	55.5
B-HCC versus NBNC-HCC	12 [3,4,26-29,31,32,35,37,38,41]	1190	B-HCC = 61.8%, NBNC-HCC = 45.5%	2.61	1.56, 4.64	< 0.001	63.1
C-HCC versus NBNC-HCC	12 [3,4,26-29,31,32,35,37,38,41]	1454	C-HCC = 69.9%, NBNC-HCC = 45.5%	3.92	2.35, 6.53	< 0.001	56.4
Vascular invasion							
B-HCC versus C-HCC	17 [3-5,26-30,34-42]	3760	B-HCC = 46.2%, C-HCC = 34.4%	1.29	0.97, 1.73	0.08	61.2
B-HCC versus NBNC-HCC	12 [3-5,26-29,35,37-39,42]	1454	B-HCC = 31.9%, NBNC-HCC = 32.9%	1.44	0.99, 2.11	0.06	37.5
C-HCC versus NBNC-HCC	12 [3-5,26-29,35,37-39,42]	1579	C-HCC = 28.7%, NBNC-HCC = 32.9%	0.99	0.62, 1.56	0.96	59.0
Intrahepatic metastases/satellite nodules							
B-HCC versus C-HCC	11 [4,26,28-31,35,37-40]	1836	B-HCC = 31%, C-HCC = 24.5%	1.23	0.87, 1.73	0.24	42.0
B-HCC versus NBNC-HCC	9 [4,26,28,29,31,35,37-39]	726	B-HCC = 30.3%, NBNC-HCC = 28.8%	1.01	0.56, 1.83	0.97	49.9
C-HCC versus NBNC-HCC	9 [4,26,28,29,31,35,37-39]	1030	C-HCC = 24.4%, NBNC-HCC = 28.8%	0.98	0.69, 1.39	0.91	23.8
Capsule formation							
B-HCC versus C-HCC	8 [4,26,27,29,30,36,38,39]	1509	B-HCC = 47.7%, C-HCC = 53.8%	0.86	0.57, 1.29	0.46	53.8
B-HCC versus NBNC-HCC	6 [4,26,27,29,38,39]	786	B-HCC = 49.3%, NBNC-HCC = 47.9%	0.96	0.55, 1.67	0.88	51.1
C-HCC versus NBNC-HCC	6 [4,26,27,29,38,39]	725	C-HCC = 52.1%, NBNC-HCC = 47.9%	1.10	0.77, 1.57	0.60	18.9
Serum AFP level (ng/ml)							
B-HCC versus C-HCC	9 [3,28-31,33,34,36,41]	1611	B-HCC = 11555.3 ± 45653.8, C-HCC = 2496.0 ± 9014.5	-52.96	-281.61, 175.69	0.65	39.5
B-HCC versus NBNC-HCC	6 [3,28,29,31,33,41]	458	B-HCC = 13927.5 ± 56323.0, NBNC-HCC = 3069.1 ± 9330.6	1385.80	-1099.05, 3870.66	0.27	86.8
C-HCC versus NBNC-HCC	6 [3,28,29,31,33,41]	1064	C-HCC = 2181.0 ± 8052.8, NBNC-HCC = 3069.1 ± 9330.6	-214.61	-714.20, 284.98	0.40	0
Survival							
5-year overall survival							
B-HCC versus C-HCC	14 [3-5,26-28,30,34-36,38,40-42]	3427	B-HCC = 51.4%, C-HCC = 52.9%	1.00	0.76, 1.31	0.99	61.9
B-HCC versus NBNC-HCC	9 [3-5,26-28,35,38,41]	1289	B-HCC = 50.2%, NBNC-HCC = 53.0%	0.68	0.44, 1.06	0.09	55.8
C-HCC versus NBNC-HCC	9 [3-5,26-28,35,38,41]	1239	C-HCC = 49.0%, NBNC-HCC = 53.0%	0.61	0.33, 1.11	0.10	75.7
5-year disease-free survival							
B-HCC versus C-HCC	13 [3,4,26,28-30,34,35,37-41]	2113	B-HCC = 32.3%, C-HCC = 25.5%	1.46	0.88, 2.41	0.14	77.8
B-HCC versus NBNC-HCC	10 [3,4,26,28,29,35,37-39,41]	860	B-HCC = 28.7%, NBNC-HCC = 49.3%	0.39	0.28, 0.53	< 0.001	33.4
C-HCC versus NBNC-HCC	10 [3,4,26,28,29,35,37-39,41]	1245	C-HCC = 26.8%, NBNC-HCC = 49.3%	0.37	0.22, 0.64	< 0.001	69.4

OR = odds ratio; WMD = weighted mean difference; CI = confidence interval; HCC = hepatocellular carcinoma; B-HCC = hepatitis B-related hepatocellular carcinoma; C-HCC = hepatitis C-related hepatocellular carcinoma; NBNC-HCC = no infection of HBV or HCV related hepatocellular carcinoma; AFP = alpha fetoprotein; ALT = alanine aminotransferase; AST = aspartate aminotransferase; T-Bil = total bilirubin; ICG R15 = indocyanine green retention rate at 15 minutes.

The mean age of patients in the B-HCC group was significantly younger than that of both the C-HCC (WMD: -10.11, 95% CI: -11.14 to -9.09, *P *< 0.001) and the NBNC-HCC groups (WMD: -10.42, 95% CI: -12.72 to -8.12, *P *< 0.001). The prevalence of male sex was higher in the B-HCC group than in the NBNC-HCC group (OR: 1.43, 95% CI: 1.10 to 1.86, *P *= 0.008). They also were more male in the B-HCC group than in the C-HCC group, although the differences were not statistically significant (*P *= 0.24).

### Liver Function

Serum aspartate aminotransferase and alanine aminotransferase levels were higher in the C-HCC group than in the other two groups. The serum total bilirubin level and indocyanine green retention rate at 15 min were higher, and the serum albumin level was lower in the C-HCC group than in the NBNC-HCC group. The platelet count was higher in the NBNC-HCC group than in the other two groups. The Child's grade A was more frequently recognized in the B-HCC group than in the C-HCC group (Table 2).

### Tumor characteristics

The mean tumor size was significantly larger in B-HCC and NBNC-HCC group than in C-HCC group (respectively: WMD: 1.32, 95% CI: 0.38 to 2.27, *P *= 0.006; WMD: -0.86, 95% CI: -1.27 to -0.45, *P *< 0.001). No significant differences were observed between B-HCC and NBNC-HCC group but NBNC-HCC group tended to have larger tumors (*P *= 0.97). The prevalence of liver cirrhosis was the highest in the C-HCC group, followed by the B-HCC group, and the NBNC-HCC group (*P *< 0.01). The incidence of vascular invasion, intrahepatic metastases/satellite nodules, tumor capsule formation, and serum AFP level, all were similar in the three groups (Table 2).

### Survival

There was a tendency toward higher 5-year overall survival rates in the NBNC-HCC group compared to those in the other two groups, although these differences were not statistically significant (Table 2).

**Figure 1:**
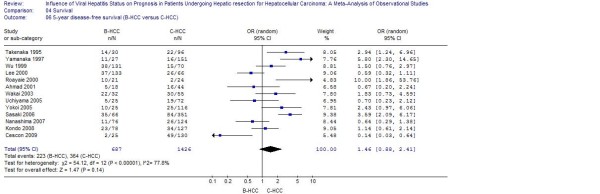
**B-HCC versus C-HCC: Results of the meta-analysis on 5-year disease-free survival**. All based on a random-effects meta-analysis.

**Figure 2:**
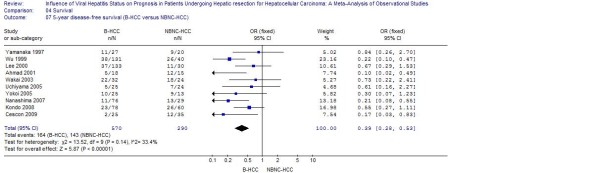
**B-HCC versus NBNC-HCC: Results of the meta-analysis on 5-year disease-free survival**. All based on a fixed-effects meta-analysis.

**Figure 3:**
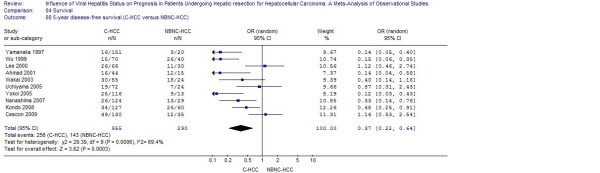
**C-HCC versus NBNC-HCC: Results of the meta-analysis on 5-year disease-free survival**. All based on a random-effects meta-analysis.

Pooled analysis of studies furnishing data found that patients with HBV or HCV infection had a worse 5-year disease-free survival when compared to patients with NBNC-HCC (respectively: OR: 0.39, 95% CI: 0.28 to 0.53, *P *< 0.001; WMD: 0.37, 95% CI: 0.22 to 0.64, *P *< 0.001) (Figure 1, 2 and 3).

Both the 5-year overall survival and disease-free survival in the B-HCC and C-HCC groups were not significantly different (Table 2).

## Discussion

HBV belongs to a family of DNA viruses called hepadnaviruses. The oncogenic potential of HBV has been attributed to its ability to integrate into host cellular DNA, which, may activate neighboring cellular genes directly to offer a selective growth advantage to the liver cells. In addition, production of hepatitis B × (HBx) protein can act as a transactivator on various cellular genes for cell growth and tumorigenesis [43]. In contrast, HCV is a positive-stranded RNA virus the genome of which does not seem to integrate into hepatocyte's genome [44]. Therefore, differences in carcinogenetic mechanisms between these viruses may affect HCC characteristics.

Most chronic HBV infections are vertical transmissions during delivery, whereas HCV infections are known to be blood-borne such as from transfusions and occurs mainly after the age of 20 years. Consequently, the mean age at occurrence of HCC is lower in B-HCC than in C-HCC. Interestingly, we also found that the mean age for patients with NBNC-HCC is significantly older than the B-HCC group. It is suspected that NBNC-HCC requires a longer time until it develops HCC [33]. The liver cirrhosis was more frequently recognized in the C-HCC group than in the B-HCC and NBNC-HCC groups. Thus, as reflected by many parameters, among the three groups, liver function was the worst in the C-HCC group.

HCC is more prevalent in men than in women, this trend is less apparent for patients with HCC unrelated to HBV. Both animal and human studies support the importance of androgen signaling in determining the male preference of HCC [45]. Increased expression and activation of androgen receptor (AR) was found in HCC and nontumorous liver tissue [46]. A recent study demonstrated that the HBx protein increased the anchorage-independent colony-formation potency of AR in a nontransformed mouse hepatocyte cell line. In addition, HBx functioned as a positive transcriptional coregulator to increase AR-mediated transcriptional activity [47]. These findings may provide a plausible explanation for the male gender preference of HBV-related HCC.

With respect to tumor factors, this study demonstrated that patients in the NBNC-HCC group had largest tumors. This was probably due to fewer NBNC-HCC patients receiving regular follow-up for the liver diseases since the two major risk factors for HCC, HBV and HCV, were negative [6,7,33]. The HCC might be discovered only when the tumor increases in size and caused subjective symptoms in the NBNC-HCC patients. The smaller tumors in the C-HCC group may be explained by the fact that C-HCC occurring at a much older age. Older age with possible comorbidities and relatively poor liver function usually preclude C-HCC patients with larger tumors from undergoing surgery [42].

In the present study, 5-year disease-free survival rates were significantly higher in the NBNC-HCC group than in the B-HCC and C-HCC groups. High rate of intrhepatic recurrence after surgical resection is the main cause of late death of patients with HCC [48]. According to point of recurrences time from the date of hepatectomy, recurrences were classified into early (≤ 2 year) and late (> 2 year) recurrences [49]. Early recurrences appear to arise mainly from intrahepatic metastases from residues of original HCC, whereas late recurrences are more likely to develop on the basis of underlying liver diseases, resulting from new carcinogenesis. It is generally accepted that virus-induced chronic inflammatory necrosis and hepatocyte necrosis might cause the hepatocytes to undergo proliferation and thus increase the occurrence of genetic aberrations, which may be the main mechanism responsible for late intrahepatic recurrence [49]. Wakai *et al*. [37] found that the cumulative probability of intrahepatic recurrence reached a plateau at 2.4 years after resection in the NBNC group, while it continued to increase steadily in the hepatitis viral groups. Thus, improved disease-free survival in the NBNC-HCC group is attributed to a low incidence of multicentric carcinogenesis, which is caused by chronic viral attack. In addition, NBNC patients maintained good liver function following the initial hepatectomy, and these biological advantages provided NBNC patients more opportunities for repeat resection of intrahepatic recurrences, which may lead to a favorable outcome [38].

Both the 5-year overall survival and disease-free survival in the B-HCC and C-HCC groups were not significantly different, indicating that influence of the viral etiology on the outcome of resection surgery in HCC patients was not obvious.

As a limitation, there are important heterogeneities between studies. There are many differences between the studies that serve as sources of heterogeneity, including variation in surgical skill, variation in perioperative and postoperative care. The other main source to the heterogeneity is NBNC-HCC group and the C-HCC group may have included patients with HBV. It was demonstrated that HBV DNA can be detected in the hepatic parenchyma of many HBsAg-negative HCC patients [50]. However, the determination of HBV DNA in liver tissue is not routinely checked during the clinical course of HCC. Given this heterogeneity, we applied a random effect model to take between study variation into consideration. This does not necessarily rule out the effect of heterogeneity between studies, but one may expect a very limited influence. Another limitation is all of data in the present study comes from observational studies. Observational studies are subject to a number of biases, including recall and selection [51]. In addition, since HCC is found commonly in China and other parts of South East Asia, most studies included in current meta-analysis were performed in Asian patients and the data cannot be extrapolated to the non- Asian population.

## Conclusions

Our meta-analysis showed HCC patients with viral infection had a poor prognosis compared to patients with negative serology. It is hypothesized that antiviral therapies would help prevent HCC recurrence by cleaning the carcinogenic soil and eliminating possibilities of novel tumorigenesis through their viral suppression and anti-inflammation action. This theory is supported by a recently published meta-analysis, in that study postoperative adjuvant antiviral therapy has a significant beneficial effect after curative treatment of HBV/HCV related HCC in terms of both survival and tumor recurrence [52]. Thus, for HCC patients with viral infections, postoperative adjuvant antiviral therapy is needed to improve the outcome.

## Competing interests

The authors declare that they have no competing interests.

## Authors' contributions

YZ participated in the design and coordination of the study, carried out the critical appraisal of studies and wrote the manuscript. LW, XS, and XS developed the literature search, carried out the extraction of data, assisted in the critical appraisal of included studies and assisted in writing up. YZ, ZZ, and BL carried out the statistical analysis of studies. All authors read and approved the final manuscript.
